# Significant Association Between Glucokinase Regulatory Protein Variants and Genetic and Metabolic Diseases

**DOI:** 10.3390/cimb47100850

**Published:** 2025-10-15

**Authors:** Ke Xu, Peng Chen, Yujing Su, Yanghui Chen, Xiuli Song, Bo Yu, Hong Wang

**Affiliations:** 1 Division of Cardiology, Department of Internal Medicine and the Genetic Diagnosis Center, Tongji Hospital, Tongji Medical College, Huazhong University of Science and Technology, Wuhan 430030, China; xuke_98@163.com (K.X.); chenpeng_088@126.com (P.C.); suyujing@hust.edu.cn (Y.S.); chenyh_c@tjh.tjmu.edu.cn (Y.C.); songxiuli_2006@126.com (X.S.); 2 Hubei Key Laboratory of Genetics and Molecular Mechanisms of Cardiological Disorders, Wuhan 430030, China

**Keywords:** GCKR variants, genetic diseases, metabolic diseases, hypertriglyceridemia, metabolic syndrome

## Abstract

As next-generation sequencing develops, there are significant associations between glucokinase regulatory protein (GCKR) variants and many diseases, especially metabolic diseases. However, there is a lack of solid descriptions and summaries of how GCKR variants lead to diseases and a lack of successful translations of drugs targeting this molecular variant. We searched literature datasets, mainly including PubMed and Web of Science, with “GCKR” or “GKRP”, “Variants”, “Hypertriglyceridemia”, “NAFLD”, and “Metabolic diseases” as the search terms. Our review firstly introduces the biological function of the *GCKR* gene and its encoding protein GKRP and then describes the *GCKR* variants in different diseases, such as hypertriglyceridemia and NAFLD, revealing that GCKR/GKPR is strongly associated with metabolic diseases. GKPR might be a potential target for T2D and other metabolic diseases. One drug for interfering with the GCK-GKRP complex has been developed and has shown its effectiveness in preclinical studies, with some possible side effects. More and more different-structured drugs should be developed to improve side effects, and more clinical trials should be carried out to determine the best intervention window and timing points to improve prognosis. Taken together, these insights show that GCKR/GKRP is significantly associated with many metabolic diseases via its complex metabolism system and is a potential target in many metabolic diseases.

## 1. Brief Introduction to Glucokinase Regulatory Protein

**Figure 1:**
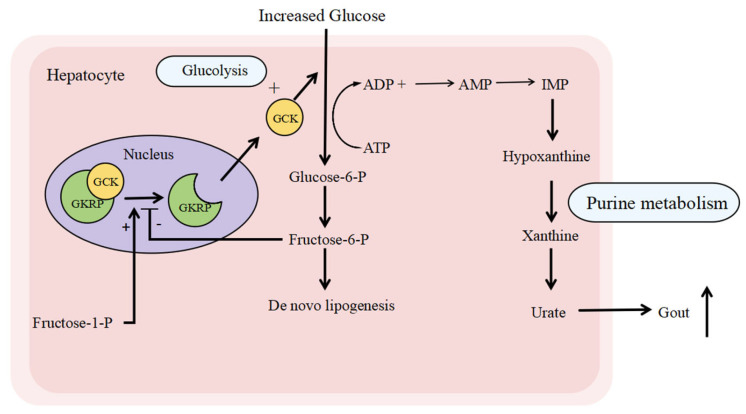
The biological functions related to GKRP. Glucokinase regulatory protein (GKRP) is located at the nucleus of the hepatocyte. When the serum glucose increases, GKRP releases GCK and enables the migration of GCK to the cytoplasm, where it converts glucose to glucose-6-P, which can be stored as glycogen or as fat (through de novo lipogenesis). Meanwhile, the enhancement of glycolysis leads to increased purine metabolism and releases more urate into the blood, causing gout. On the other hand, fructose 1-phosphate (fructose-1-P) is a natural disruptor of the GKRP-GCK complex, whereas fructose 6-phosphate (fructose-6-P) stimulates its binding. Abbreviations: GKRP, glucokinase regulatory protein; GCK, glucokinase; ATP, adenosine triphosphate; ADP, adenosine diphosphate; AMP, adenosine monophosphate; IMP, inosine monophosphate.

Glucokinase regulatory protein (GKRP), encoded by the *GCKR* gene, is biologically and genetically involved in key metabolic pathways [1]. Located in the nucleus of hepatocytes, GKRP binds to and inhibits glucokinase (GCK), thereby helping regulate blood glucose levels by controlling glucose uptake and storage (Figure 1). GCK acts as a critical regulator of glucose disposal and storage in both the liver and pancreatic beta-cells. It responds to rising blood glucose concentrations by initiating a cascade that leads to insulin secretion from beta-cells and subsequent hepatic glucose uptake and storage. As an inhibitor of GCK—an essential enzyme that phosphorylates glucose to glucose-6-phosphate (the first step of glycolysis)—GKRP plays a central regulatory role [2]. Notably, GKRP is expressed exclusively in the liver, despite the abundance of GCK in the pancreas, suggesting that GKRP modulates glucose and lipid metabolism specifically in the liver rather than the pancreas [3,4]. Under fasting or hypoglycemic conditions, GKRP binds GCK and sequesters it in the nucleus, limiting the conversion of glucose to glucose-6-phosphate and thereby increasing blood glucose levels. This nuclear sequestration of GCK by GKRP suppresses hepatic glycolysis, making more glucose available for other organs. In contrast, under high-glucose conditions, GKRP’s affinity for GCK decreases, releasing GCK into the cytoplasm, where it promotes glycogen synthesis and de novo lipogenesis [5,6,7]. The interaction between GKRP and GCK is further modulated by metabolic intermediates: fructose-6-phosphate (F6P) enhances their binding and suppresses glycolysis, while fructose-1-phosphate (F1P) inhibits this interaction [2]. Enhancing GKRP function may lower blood glucose levels, offering a potential therapeutic strategy in diabetes [8]. Moreover, GKRP helps stabilize GCK, as GCK degradation is observed in GKRP-knockout models [9,10]. Overall, GKRP critically regulates glucose metabolism by controlling the binding, localization, and activity of GCK.

With the completion of the Human Genome Project and advances in next-generation sequencing, numerous studies have identified significant associations between GCKR variants and metabolic diseases, including type 2 diabetes, non-alcoholic fatty liver disease (NAFLD), and familial hypertriglyceridemia [11,12,13]. This review summarizes the functional impacts of GCKR genetic variants on metabolism and discusses potential therapeutic strategies targeting GKRP.

## 2. Glucokinase Regulatory Variants and Hypertriglyceridemia

Hypertriglyceridemia, a common form of hyperlipidemia, is typically defined as a fasting serum triglyceride level of 150 mg/dL (1.7 mmol/L) or higher, according to the American Heart Association (AHA) 2011 guidelines [14,15,16]. It represents the most prevalent type of dyslipidemia in the general population. Data from the NHANES 2003–2006 survey indicate that approximately 53% of U.S. adults have dyslipidemia, among whom 30% exhibit elevated serum triglycerides (>1.7 mmol/L), while 27% have high serum low-density lipoprotein (LDL) cholesterol levels [17]. Excess lipid deposition in various organs can lead to conditions such as fatty liver disease and pancreatitis [17]. Moreover, hyperlipidemia is recognized as a major risk factor for cardiovascular diseases, including atherosclerosis, myocardial infarction, and heart failure [18,19]. Hypertriglyceridemia arises from factors such as a sedentary lifestyle, poor dietary habits, and genetic variants. Numerous genome-wide association studies (GWASs) and whole-exome sequencing (WES) analyses have identified several genes such as *Apolipoprotein A5 (APOA5), Lipoprotein lipase (LPL), Apolipoprotein C2 (APOC2), Familial lipase maturation factor 1 (LMF1), and Glycosylphosphatidylinositol-Anchored High-Density (GPIHBP1),* variants of which lead to hypertriglyceridemia [20,21,22], while variants of *Apolipoprotein B, (APOB), Low-density lipoprotein receptor (LDLR), and Proprotein Convertase Subtilisin/Kexin Type 9 (PCSK9)* lead to hypercholesteremia [23,24,25,26].

**Figure 2:**
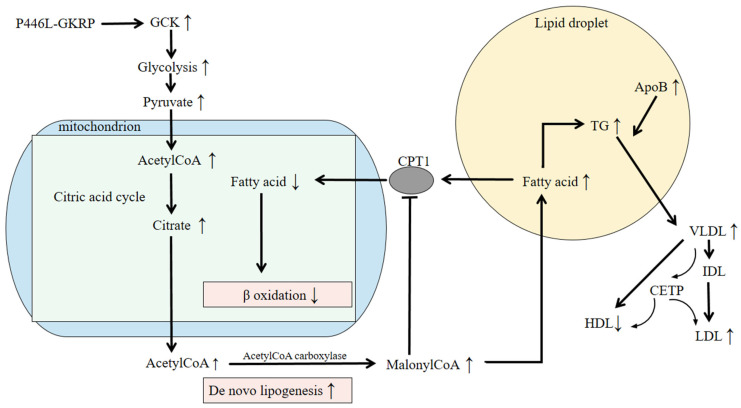
The pathogenic mechanism of P446L-GKRP. The GCKR variant rs1260326 leads to GKRP P446L and promotes de novo lipogenesis, leading to hepatic fat deposition and hypertriglyceridemia. P446L-GKRP disturbs the production of the GKRP-GCK complex and enables GCK to migrate into the cytoplasm. The increased glycolysis produces more pyruvate, which results in increased acetylCoA production. AcetylCoA participates in the citric acid cycle, leading to higher amounts of citrate. Excessive citrate is then used to synthesize fatty acids. MalonyCoA produced in the process of de novo lipogenesis hampers the oxidation of fatty acids (FAs) by inhibiting CPT1, a mitochondrial FA transporter, which leads to liver fat deposition. Meanwhile, increased fatty acid formation results in TG accumulation and increased hepatic fat, stimulating the production of ApoB VLDL particles, which are degraded into IDL and LDL and release more TGs. HDL and LDL particles exchange cholesteryl ester (CE) for TGs with VLDL particles, mediated by CETP. The above may result in higher serum triglyceride levels and lead to hypertriglyceridemia. Abbreviations: GKRP, glucokinase regulatory protein; GCK, glucokinase; CoA, coenzyme A; CPT1, Carnitine palmitoyltransferase1; TG, triglyceride; ApoB, apolipoproteinB; VLDL, very-low-density lipoprotein; IDL, intermediate-density lipoprotein; LDL, low-density lipoprotein; CETP, cholesteryl ester transfer protein; HDL, high-density lipoprotein.

Among these, GCKR variants, initially recognized for their role in glucose metabolism, have attracted increasing attention. Although GKRP was first characterized as a glycolysis regulator, recent genomic studies have strongly linked its variants to triglyceride levels. Stable isotope studies suggest that hepatic fat accumulation may enhance the synthesis of very-low-density lipoprotein (VLDL). Loss-of-function variants in GCKR may reduce its binding affinity for glucose and promote hepatic fat accumulation, thereby increasing VLDL secretion [27,28]. Fat accumulation in the liver also stimulates the production of apolipoprotein B (APOB), a key component of VLDL particles [29]. After secretion, VLDL is progressively converted to intermediate-density lipoprotein (IDL) and then to LDL. Cholesteryl ester transfer protein (CETP) facilitates the exchange of triglycerides and cholesteryl esters among VLDL, LDL, and HDL particles. This process can result in elevated LDL and reduced HDL levels, ultimately contributing to hyperlipidemia (Figure 2).

Hyperlipidemia, especially hypertriglyceridemia, is strongly associated with GCKR variants according to current studies. The non-synonymous variant *rs1260326* (p. Pro446Leu) and the intronic SNP *rs780094* are two common variants [30,31].

The common variant rs1260326 has been detected in many GWASs. The fine mapping of the GCKR locus revealed that SNP rs1260326 exhibited the strongest correlation with triglyceride concentrations and showed a strong linkage disequilibrium [31,32]. Furthermore, several GWASs on diabetes and hyperlipidemia have also found this variant. According to the ClinVar database, the global minor allele frequency (MAF) of rs1260326 is almost 0.3, indicating that roughly one in three individuals carries this variant. Rees reported that rs1260326 significantly downregulated the expression of GKRP and its interaction with glucokinase in the absence of any intervention, similar to when fructose-1-phosphate (F1P) and fructose-6-phosphate (F6P) were administered [33]. In addition, no difference was observed in the direct affinity between F1P and F6P. Zelent et al. found that glucose, fructose 1-phosphate, and glucokinase activators decreased the affinity of GK for GKRP and increased the binding cooperativity. These findings reflected the disruption of the GK–GKRP complex, while TF-based biophysical analysis revealed that GKRP-P446L might impair the GCK-GKRP complex and nuclear storage [34]. This may explain the inverse change in serum glucose and triglyceride in the fasting state. Despite the increasing number of GCKR variants discovered via NGS, rs1260326 continues to play a pivotal role, prompting additional research into the functions and phenotypes of GCKR variants.

Compared with rs1260326, rs780094 is less researched due to its position on an intron. This variant correlates with the expression of GCKR and the occurrence of hyperlipidemia, with a minor allele frequency (MAF) ranging from 0.2 to 0.4 according to various databases. Moreover, the SNP rs780094 is associated with higher triglyceride, apoB, and CRP levels but not LDL or HDL cholesterol levels [31]. The loci located in a liver-specific enhancer and haploid deficiency contribute to associated traits. In addition, Forkhead box A2 (FOXA2) combines with the enhancer and elevates H3K27Ac levels to promote GCKR transcriptional activity. In the rs780094 variant, the T allele replaces the C allele, which may result in decreased binding to FOXA2 and GCKR transcriptional activitiy [35].

Detection and functional analysis of the above two variants have provided mechanistic insights into the pathways affected by the GCKR variations. In addition to the above common variants, other rare variants have also been detected in genome studies. Johansen identified rare GCKR variants in 438 individuals with hypertriglyceridemia compared with the control cohort [32]. Rees et al. also extended the atlas of GCKR variants and presented a model for interpreting the clinical significance of rare genetic variants in common diseases in 2012 [36]. They identified 10 novel rare coding variants by sequencing the exome of GCKR in 800 coronary atherosclerosis patients from the ClinSeq cohort [36]. The following functional analysis of the 18 rare non-synonymous GCKR variants was conducted by biological experiments and bioinformatic prediction, but the lack of consistency between the two analyses emphasized the low predictive value of rare GCKR variants and the complex heritability of lipid traits [33]. Western blot analysis was performed to study different expression levels of GKPR, and homogenous time-resolved fluorescence and microscale thermophoresis were used to compare the affinity of recombinant WT and variant GKRP for GCK, F1P, and F6P. Subsequently, the Ribbon model of the F1P-bound form of human F1P was run. The inconsistent results between bioinformatic predictions and functional assays for rare variants underscore the challenges in their clinical interpretation. This suggests that the pathogenicity of rare GCKR variants cannot be reliably predicted in silico and requires functional validation. Consequently, the clinical utility of testing rare GCKR variants in hypertriglyceridemia remains limited until more robust variant-specific evidence is available. This stands in contrast to the well-established association of common variants like rs1260326.

In a recent study, Ford et al. used adenoviral vectors for human or mouse GCKR. P446L was transfected in hepatocytes and diet-challenged P446L mice, revealing that the diet-challenged P446L mice developed several traits found to correspond to the rs1260326 locus on chromosome 2, including raised blood cholesterol, lower blood glucose, and low-er liver glucokinase and GKRP, but not raised blood triglycerides [37]. This discrepancy with human association studies might be attributed to differences in murine and human lipid metabolism and the specific dietary challenge employed. The different genotypic milieu between human and mice and the instability of the model system should not be ignored. In addition, the different GKRP perturbation in the model system should be considered; AAV infection is acute and short-term, while the GCKR variant causes injury from birth. Maybe an earlier infection time point could be tried, or GCKR point mutation models might be a better choice. Further research is needed to reconcile these findings.

According to many studies and case reports, GCKR variants do not cosegregate with triglycerides in hypertriglyceridemic pedigrees [33,38]. It is theorized that hereditary disease may be caused by the interaction between different gene mutations or other risk factors, which may provide evidence for Knudson’s two-hit hypothesis [39]. In addition to different gene mutations, the different loci of the same gene may contribute to disease occurrence as well. A study in 2016 reported that a patient carried three distinct GCKR mutation sites and suffered from severe hyperlipidemia following her pregnancy [38]. These findings suggest that primary familiar hypertriglyceridemia may arise from a complex genetic basis, as they are often caused by the interaction between several different gene variants or environmental factors instead of an individual gene variant [40,41].

## 3. Glucokinase Regulatory Protein and Non-Alcoholic Fatty Liver Disease

Non-alcoholic fatty liver disease (NAFLD) is a common chronic liver condition worldwide, with a prevalence of approximately 25% in the adult population [42]. It is recognized as a hepatic manifestation of metabolic syndrome and is frequently associated with obesity, insulin resistance, and dyslipidemia. NAFLD encompasses a spectrum ranging from simple hepatic steatosis to inflammation and hepatocyte ballooning, primarily resulting from non-secondary hepatic fat accumulation [43]. In recent years, genome-wide association studies (GWASs), whole-exome sequencing (WES), candidate gene analyses, and family-based studies have indicated a significant genetic component underlying NAFLD. Family studies suggest that genetic factors account for at least 38% of the variability in hepatic fat content and NAFLD susceptibility [44,45]. Several gene loci have been implicated, including the GCKR variants rs1260326 (P446L) and rs780094 [46,47].

Notably, GCKR mRNA expression is significantly reduced in patients with severe hepatic steatosis compared to those with mild steatosis [48], highlighting the need for further research to identify pathogenic GCKR variants and elucidate their role in NAFLD pathogenesis. Both in vitro and in vivo studies suggest that the rs1260326 polymorphism impairs the inhibitory function of GKRP toward GCK, leading to enhanced hepatic glucose uptake, accelerated de novo lipogenesis, and ultimately increased hepatic fat accumulation [3,41,49,50]. Meanwhile, rs780094 is located within a liver-specific intragenic enhancer that modulates GCKR expression in a haplotype-dependent manner [35]. Strong linkage disequilibrium between rs1260326 and rs780094 suggests that these variants may collectively increase NAFLD risk [31]. A large-scale meta-analysis involving 16,927 participants (5115 cases and 11,812 controls) for rs780094 and 11,233 participants (2238 cases and 8995 controls) for rs1260326 further confirmed their significant association with NAFLD, with odds ratios of 1.20 (95% CI: 1.11–1.29) for rs780094 and 1.32 (95% CI: 1.22–1.42) for rs1260326 [30].

## 4. Glucokinase Regulatory Protein and Metabolic Syndrome

Although NAFLD is considered a component of metabolic syndrome, GCKR variants have also been significantly associated with a broader range of metabolic abnormalities. These variants influence an extensive array of metabolites involved in carbohydrate, fatty acid, amino acid, purine, and lipid metabolism (Figure 1 and Figure 2) [51,52,53,54,55], as summarized in the review by Brouwers et al. [28]. A meta-analysis of 40,000 subjects showed that homozygous carriers of the rs780094 variant had 1.9% lower plasma glucose levels but 13% higher triglyceride concentrations compared to non-carriers [31]. Interestingly, GCKR variants exhibit a distinct relationship with type 2 diabetes (T2D) relative to other metabolic disorders. For instance, some GCKR variants that are protective against T2D have been positively associated with elevated branched-chain amino acids such as isoleucine and leucine, which may conversely increase T2D risk [56,57,58]. Additionally, genetic studies have linked GCKR variants to higher levels of C-reactive protein (CRP) and coagulation factors such as protein C and factor VII [59,60,61,62]. The relationship between GCKR variants and CRP may be partly mediated by NAFLD. CRP, an acute-phase inflammatory protein, is produced in response to IL-6 [63]. Elevated IL-6 and CRP levels are common in obesity, and although direct evidence linking GCKR variants to obesity is limited, this pathway warrants further investigation [64]. One report suggested that CRP levels are more closely associated with hepatic fat content than with obesity itself, implying that NAFLD may contribute to increased CRP levels [65].

## 5. Glucokinase Regulatory Protein and Diabetes Mellitus

Diabetes mellitus is a complex disease influenced by multiple genetic and environmental factors, complicating the identification of its underlying causes and the development of effective therapies. To date, genome-wide association studies (GWASs) and other genetic approaches have identified several susceptibility loci, including *peroxisome proliferator-activated receptor gamma (PPARG), glucagon-like peptide 1 receptor (GLP1R), and transcription factor 7-like 2 (TCF7L2)* [66,67,68]. Some of these discoveries have already translated into therapeutic targets; for example, PPARG is the target of insulin-sensitizing thiazolidinediones, and small-molecule GLP1R agonists have gained widespread clinical use [69,70]. Pathogenic variants in GCK are known causes of maturity-onset diabetes of the young (MODY) [71,72]. Functional studies indicate that these variants reduce glucose affinity, impairing beta-cell insulin secretion and hepatic glycogen storage [73,74]. These findings support the potential utility of GCK activators in diabetes treatment, though their use is limited by a significant risk of hypoglycemia. To overcome this limitation, Lloyd et al. (2013) developed a small molecule designed to disrupt the GCK–GKRP interaction [75]. Since GKRP is expressed exclusively in the liver, this compound selectively activates hepatic GCK without affecting pancreatic beta-cells, thereby lowering fasting glucose levels with a reduced risk of hypoglycemia [76]. Using a cell-free high-throughput screening approach, the team identified a molecule that binds GKRP and promotes GCK translocation from the nucleus to the cytoplasm in primary rat hepatocytes [75]. The compound effectively reduced serum glucose levels in hyperglycemic rats but not in normoglycemic controls [75]. Subsequent studies in diet-induced obese C57BL/6 mice, as well as ob/ob and db/db models, yielded consistent results [77]. While these findings highlight the therapeutic potential of targeting the GCK–GKRP complex, potential adverse effects must be carefully considered. Studies suggest that loss-of-function GCKR variants may disrupt lipid homeostasis over time, indicating that pharmacological inhibition of GKRP could perturb lipid metabolism [10,78]. The indications and contraindications of such a drug therefore require thorough evaluation. In addition, the relationship between GCKR variants and cardiovascular outcomes remains unclear. Although rare loss-of-function GCKR variants do not consistently cosegregate with hypertriglyceridemia, their influence on lipid levels should not be overlooked [33]. For instance, while one study reported that the rs780094 variant may protect against cardiovascular disease, other large-scale studies have reached conflicting conclusions [79,80,81]. In one follow-up analysis, GCKR polymorphism was associated with an increased cardiovascular risk, though a significant association was observed only for ischemic cardiomyopathy after adjustment for lipid levels and T2D status—a finding that may limit its reliability [81].

A notable paradox arises between the goal of pharmacologically inducing GKRP loss-of-function to lower blood glucose and the adverse metabolic profile associated with genetic GCKR loss-of-function [82]. This discrepancy may stem from the chronic nature of genetic deficiency versus the acute pharmacological effect, as well as the complex interplay of metabolic pathways involved [83]. A lower dosage and extended treatment regimen may help mitigate side effects, though large-scale clinical trials will be essential to define the therapeutic window and optimal dosing. In summary, further clinical studies are needed to evaluate the efficacy, safety, and overall risk–benefit profile of GKRP-targeting glucose-lowering agents. Genetic screening may also play a future role in personalizing treatment and identifying patients most likely to respond safely to this approach.

## 6. Limitations and Future Studies

Although numerous studies have established the association between GCKR variants and metabolic diseases, confirming both the pathogenicity of these variants and the therapeutic potential of targeting GKRP, translational progress into clinical practice remains limited. The development of small molecules that disrupt the GCK–GKRP complex represents a promising therapeutic strategy, potentially offering a novel approach to T2D management without the risk of systemic hypoglycemia. Such targeted agents echo the success of other genetics-driven therapies, such as PCSK9 inhibitors for lipid management [84,85]. However, the potential side effects of GKRP modulation—particularly on lipid homeostasis—must be carefully addressed through further preclinical and clinical studies. Notably, emerging multi-target agents such as GLP-1R/GCGR dual agonists are already under investigation for metabolic and liver diseases [86], highlighting the growing interest in pathway-based therapies. Given the complex role of GKRP within the metabolic system, minimizing side effects—particularly on lipid metabolism—poses a significant challenge. Future research should focus on identifying optimal therapeutic windows, including appropriate dosing and treatment timing, through well-designed clinical trials. Additionally, artificial intelligence could aid in designing more precise drug structures, while alternative therapeutic platforms such as antisense oligodeoxynotides (ASOs) or adeno-associated virus (AAV)-based approaches may offer new avenues. Concurrently, further genetic studies on GCKR variants across different diseases will help clarify the clinical significance of GKRP and strengthen the evidence base for its therapeutic targeting.

## 7. Conclusions

The GCKR gene and its encoded protein, GKRP, play essential roles in multiple metabolic pathways, including glucose, lipid, and uric acid metabolism [87,88]. Both common and rare GCKR variants have been linked to hypertriglyceridemia, NAFLD, and type 2 diabetes, underscoring their broad impact on human metabolic health. Among these, the rs1260326 variant stands out due to its strong genetic association and functional relevance across a spectrum of metabolic diseases. With advances in next-generation sequencing and increasing numbers of family-based and population genetic studies, more GCKR variants are likely to be identified, enabling deeper functional characterization and clearer mechanistic insights. With continued research, GKRP-targeted interventions may eventually find a place in clinical practice, offering new options for patients with metabolic disorders.

## 8. Methods in the Search Strategy

The literature datasets mainly included PubMed and Web of Science, and the search terms included “GCKR” or “GKRP”, “Variants”, “Hypertriglyceridemia”, “NAFLD”, and “Metabolic diseases”. The inclusion criteria were that (1) the article comprised original research or a case report; (2) there was a comprehensive meta-analysis; (3) it described and investigated significant associations between GCKR variants and metabolic diseases. The exclusion criteria were that the article (1) showed a lack of solid variants and sequencing evidence; (2) was published before the 1980s.

## Data Availability

The data presented in this study are available on request from the corresponding author.
